# Multi-staged development and pilot testing of a self-assessment tool for organizational health literacy

**DOI:** 10.1186/s12913-023-10448-0

**Published:** 2023-12-13

**Authors:** Izumi Klockmann, Leonie Jaß, Martin Härter, Olaf von dem Knesebeck, Daniel Lüdecke, Johanna Heeg

**Affiliations:** 1 Department of Medical Sociology, Center for Psychosocial Medicine, University Medical Center Hamburg-Eppendorf, Martinistraße 52, 20246 Hamburg, Germany; 2 Department of Medical Psychology, Center for Psychosocial Medicine, University Medical Center Hamburg-Eppendorf, Martinistraße 52, 20246 Hamburg, Germany

**Keywords:** Organizational health literacy, Health literacy responsiveness, Health care organizations, Self-assessment tool, Tool development, Health literacy, Organizational development, Patient-centered communication

## Abstract

**Background:**

Until now a comprehensive, consensus-based tool that can be used by a variety of health care organizations for assessing their organizational health literacy (OHL) is not available. Therefore, we aimed to develop and test a literature- and consensus-based self-assessment tool.

**Methods:**

The study is based on a scoping review that was previously published by the authors. For the development of the self-assessment tool, the criteria identified in the literature were synthesized with criteria gained through group discussions with representatives of different types of health care organizations (N = 27) all based in Hamburg (Germany). Consensus on the criteria was reached by conducting a Delphi process (N = 22). A review by the project’s patient advisory council was included in the process. The self-assessment tool was converted into an online tool and refined by a pretest. Finally, the online survey was piloted (N = 53) and the reliability and item loadings for each scale were analyzed.

**Results:**

In total, 77 criteria (items) characterizing a health literate health care organization were developed and grouped into five main categories (scales): (1) “easy access and navigation”, (2) “integration, prioritization, and dissemination of OHL”, (3) “qualification, quality management, evaluation, and needs assessment”, (4) “communication with target groups”, and (5) “involvement and support of target groups”. The results of the online survey showed that the tool is suitable for assessing an organization’s status quo on OHL. The psychometric analysis showed good to excellent internal consistency. Item analyses of the developed self-assessment tool was satisfactory.

**Conclusions:**

We were able to define a set of 77 items to characterize OHL, which were integrated into a new, comprehensive, and consensus-based self-assessment tool to identify areas for improvement. We found evidence that the self-assessment tool, based on the identified criteria, consists of the assumed five scales. Further research should analyze the validity of the self-assessment tool on a higher detail level.

**Supplementary Information:**

The online version contains supplementary material available at 10.1186/s12913-023-10448-0.

## Background

Organizational health literacy (OHL) refers to the ability of a health care organization to “make it easier for people to navigate, understand, and use information and services to take care of their health” [1]. In comparison to the concept of individual health literacy, which “entails people’s knowledge, motivation and competences to access, understand, appraise, and apply health information” [2]. OHL focuses on the responsibilities and scope of action on the side of the organizations in providing health care to people. It describes organizations’ competences to support patients’ orientation and navigation in the health care system and to empower patients to understand and deal with health-related information. This also includes staff-related competences, as the staff themselves must also be able to understand the health-related information provided by the organizations and communicate it in an intelligible way [3, 4].

In order to make the concept of OHL understandable and implementable, a scientifically based and easy-to-use self-assessment tool which is based on a clear framework of OHL and applies to different types of health care organizations is not yet available. So far, there are only a few tools available. However, these tools primarily focus on specific health care organizations such as hospitals [4–7], pharmacies [8], primary care practices [9–12], or organizations belonging to the community sector [13]. Other tools compose of widely formulated criteria [6] that offer a general overview of OHL but do not define OHL on a more detailed level easy to translate into precise steps of action. The advantages of a generic assessment tool include that different types of organizations can be compared and that organizations can also be examined for which there is no specific tool available.

To address this research gap, our project “OHL-HAM - Organizational Health Literacy in the Hamburg Metropolitan Area”, funded by the German Federal Ministry of Education and Research, aimed to develop and test a literature- and consensus-based self-assessment tool for OHL. This study builds on the current state of the art of OHL criteria that were identified through a scoping review published by the authors prior to this study [14]. The scoping review identified six main categories of OHL criteria: (1) “communication with service users”, (2) “easy access and navigation”, (3) “integration and prioritization of OHL”, (4) “assessments and organizational development”, (5) “involvement and support of target groups”, and (6) “information and qualification of staff” [14]. The presented study expands and refines the gathered OHL criteria and poses the following research questions: First, which criteria are required for a tool to measure OHL? And second, how suitable and applicable is the developed tool to measure OHL?

## Methods

The study is divided into two phases. During the tool development phase, criteria characterizing health literate health care organizations were gathered, categorized, revised, and converted into an online self-assessment tool. In the second phase, the self-assessment tool was piloted as an online survey and tested using psychometric analysis.

### Tool development

The development of the tool builds on the scoping review that captured the current state of the art of OHL criteria [14] and is based on a combination of qualitative and quantitative methods: qualitative group discussions, a Delphi process, and a pretest of an online survey.

## Group discussions

To include the perspective of professionals operating in health care organizations, we conducted group discussions [15, 16]. In total, 27 representatives of 24 health care organizations that differed in type and services were recruited from Hamburg (Germany) to participate. The organizations were clustered into four group discussions that were as homogeneous as possible regarding the type of their organization (see Table 1).

**Table 1:** (Number of) Organizations and participants in group discussions

Type of organizations	N organizations	N participants
Group 1: Professional organizations, chambers, associations, corporations, and authorities	5	7
Group 2: Cost bearers and health insurance companies	6	7
Group 3: Patient-centered organizations, patient representatives, and self-help organizations	5	5
Group 4: Providers and clinics	8	8
Total	24	27

The group discussions were conducted as online workshops from 09/21/2020 to 10/21/2020. The participants were provided with a brief introduction to the concept of OHL which was mainly based on Brach et al. [1] and Trezona et al. [17]. The participants were asked to discuss the following question: “What does a health care organization need to have or do to help people find, understand, and use health information and services?”. The group discussions were recorded and transcribed. In order to extract OHL criteria, qualitative content analysis was used to examine the transcripts. In particular, the technique of summarizing content analysis using inductive category formation following Mayring [18] was adopted. Coding and categorizing were carried out by one researcher (LJ). Consultation and revision of the categories took place in collaboration with a second researcher (IK). These phases were conducted in multiple loops. All participants provided informed consent prior to participating in this study.

### Synthesis

The extracted criteria from the group discussions were merged with those gained through the scoping review into newly formulated criteria. Redundancies among the criteria were reduced by synthesizing those with similar content. Three researchers (DL, IK, LJ) sorted and categorized the criteria into main and sub-categories resulting in the first version of the self-assessment tool.

### Delphi study

The first draft of the self-assessment tool was converted into an online survey as part of a Delphi study to select the most important criteria and get feedback regarding their phrasing and scope of the content. The participants of the group discussions as well as additionally recruited representatives from other health care organizations were asked to rate each criterion based on its importance concerning their organization. The final sample of the Delphi study consisted of 22 respondents all representing different organizations. Respondents could choose one of the following answers: “important”, “rather important”, “somewhat important/partly important”, “rather unimportant”, “unimportant”, “don’t know”, and “no answer”. Participants were able to comment on the items and make suggestions for changes. In addition, a patient advisory council consisting of three women commented on the draft of the self-assessment tool as part of the Delphi study. Based on Fitch et al. [19] a Delphi process was used to decide on the (lack of) consensus or on inconclusive decisions on criteria. Consensus was reached when no more than two ratings deviated from the median score by ≥ 1.5 points. Lack of consensus was defined as three or more ratings that occurred in each tail of the rating scale (rating of one or two for the lower tail, four or five for the higher tail). When ratings did not meet the criteria for consensus or lack of consensus they were considered inconclusive [19]. Results and feedback were incorporated and a new draft of the self-assessment tool underwent a second, internal feedback loop. The field time of the Delphi study lasted from 04/15/2021 to 06/27/2021. Based on the results of the Delphi process, a final draft of the self-assessment tool with five scales was developed. The five-scale structure of the self-assessment tool was coherent with the results from the scoping review and the qualitative analysis of the group discussions.

### Pretest

The pretest aimed to improve the comprehensibility, usability, and scope of the self-assessment tool. The project’s patient advisory council, scientific colleagues, and the members of the project team were invited to test the online tool. Participants were asked to note difficulties in understanding the questionnaire items and instructions as well as issues with technical aspects. The feedback was incorporated and resulted in the final version of the OHL self-assessment tool. The pretest was carried out between 08/20/2021 and 09/17/2021.

### Pilot tool testing

#### Online survey

The online survey aimed to test the developed self-assessment tool with a convenience sample of health care organizations and get a better understanding of the structure and reliability of the tool. Based on a typology of health care organizations by the German Federal Ministry of Health [20], an online search was conducted to identify organizations eligible for the sampling process. In total, 48 of 134 contacted organizations agreed to either participate in the survey themselves and/or forward the invitation. In addition, 37 organizations already involved in the study were invited to participate and/or forward the invitation. The organizations acting as multipliers forwarded the survey link, for example, to their member organizations or individual colleagues or published the link and description of the survey on their website or in their newsletter.

The first part of the online survey consisted of items regarding organizational characteristics, i.e. region, and target groups. The second part contained the items (criteria) of the developed OHL self-assessment tool. In this section, participants were asked the following question for each item: “To what extent are the following aspects regarding the access to the organization and its services currently being implemented?” The scale was labeled with: “not at all”, “a little”, “somewhat/partly”, “mostly”, “completely”, “not applicable”, “don’t know”, and “no answer”. The field time of the online survey lasted from 09/22/2021 to 11/30/2021. One additional case was added later on (08/09/2022). The final sample size consisted of N = 53 organizations.

### Statistical analyses

For descriptive analyses, data distribution (mean, standard deviation, median, etc.) was calculated with Stata (Stata/SE 15.1 for Windows).

Psychometric analysis was conducted as an exploratory analysis due to the small sample size. We investigated the structure of the self-assessment tool that was derived from the development phase. Since the structure was based on the previous results from the scoping review and the qualitative analysis of the group discussions, and due to the small sample size, we decided not to derive the factor structure empirically using quantitative methods (e.g., principal component analysis or confirmatory factor analysis). Additionally, for the same reason, the psychometric analysis was only applied to the main scales of the self-assessment tool and not to its subscales. The structure and reliability of the self-assessment tool were analyzed by calculating various measures. Floor and ceiling effects for each scale were determined by calculating the proportions of participants in the lower or upper 10% of each score [21]. Floor or ceiling effects larger than 15% were considered statistically significant and indicated poor discrimination of a scale [22]. Moreover, the difficulty for each item was calculated to indicate floor (item difficulty < 0.2) or ceiling (item difficulty > 0.8) effects per item, which means items had poor discrimination if these thresholds were exceeded [23]. We also reported the item discrimination (corrected item-total correlations for each item of a subscale with the subscale’s remaining items), where acceptable values range from 0.4 to 0.7 [23]. Cronbach’s alpha was calculated for each scale as well as for the total score. We followed common conventions to interpret Cronbach’s alpha values (α < 0.5 = unacceptable, 0.5 < α < 0.6 = poor, 0.6 < α < 0.7 = questionable, 0.7 < α < 0.8 = acceptable, α > 0.8 = good or excellent) [24]. The psychometric analyses were conducted with R, Software version 4.2.1 (R Core Team, 2022) using the packages “performance” [25] and “parameters” [26].

## Results

### Tool development

#### Group discussions

24 organizations with a total of 27 representatives participated in four group discussions implemented as online workshops. The representatives were mostly employees in management positions. Criteria characterizing health literate health care organizations were formulated and discussed. Summarizing content analysis of the transcripts resulted in a three-leveled code system containing 52 unique criteria clustered into seven main categories. Some criteria were subsumed directly under the main categories whereas other criteria were again grouped into subcategories.

#### Synthesis

The 490 criteria extracted in the scoping review that overlapped in content were merged with the 52 unique criteria gathered in the group discussions, to form a pool of 542 criteria. These criteria were paraphrased and reduced to unique criteria. The synthesis of both sets of criteria resulted in 75 criteria clustered into 17 subcategories and five main categories. Of those 75 criteria, 44 came from both the scoping review and the workshops, three criteria originated from the workshops only and were not found by the scoping review, while the remaining 28 criteria were identified exclusively via the scoping review.

#### Delphi study

Analyses in the course of the Delphi study did not identify a pattern regarding the importance of different criteria by type of organization. Overall, there was great consensus on the criteria. Several respondents gave feedback via open text fields that they would consider all of the criteria as being important and that the real challenge would be to realize or fund them with resources. Due to content-related feedback on the criteria, seven criteria were rephrased, three criteria were split up into two criteria each, and one criterion was moved to a different main category. Three criteria were dropped and one criterion was added. Further adaptations to the tool were made following the internal revision by project members. These adaptations consisted of the exclusion of one criterion, rephrasing of 12 criteria, division of two into four criteria, and merging four criteria into two. Following these adjustments, a set of 75 criteria (5 main categories, 17 subcategories) was converted into the preliminary self-assessment tool with 5 scales, 17 subscales, and a total of 75 items.

#### Pretest

Two members of the project’s patient advisory council, two colleagues, and two student research assistants participated in the pretest. After running the survey pretest, three items were rephrased and two further items were split into two items each. 77 items remained in the final version of the self-assessment tool.

#### Final self-assessment tool

The consecutive steps of the tool development resulted in the final self-assessment tool comprising 77 items grouped into five scales and 17 subscales (see Table 2 and Additional file 1).

**Table 2:** Number of subscales and items per scale in the final OHL self-assessment tool

Scale	N subscale	N items
1) Easy access and navigation	3	16
2) Integration, prioritization, and dissemination of organizational health literacy	4	11
3) Qualification, quality management, evaluation, and needs assessment	2	17
4) Communication with target groups	6	25
5) Involvement and support of target groups	2	8
Total	17	77

**Fig. 1:**
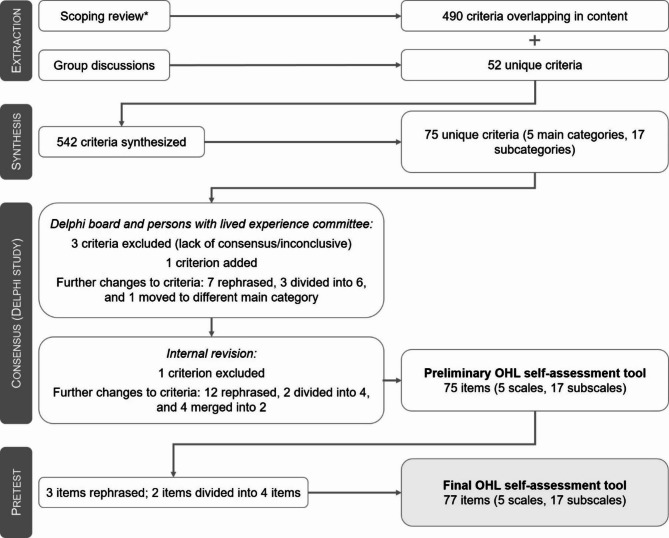
Flowchart of the development process of the OHL self-assessment tool * Scoping review by the authors [12]

The development of the OHL self-assessment tool is summarized in the flowchart below (Fig. 1).

### Pilot tool testing

#### Sample description

The final sample of 53 organizations consisted of 13 organizations that had already been involved earlier in the study, 39 organizations included through sampling, and one organization that was recruited later on in the study. The organizations were either exclusively based in the city of Hamburg (n = 46), in the city of Hamburg and other regions (n = 4), in the metropolitan region of Hamburg (n = 1), or outside of this region (n = 2). Target groups differed among the organizations. While most organizations address people who are affected themselves (e.g., patients), organizations that (exclusively) address their relatives are also part of the sample (see Table 3).

**Table 3:** Target groups of organizations in the sample

Target groups	N organizations
People who are affected themselves (e.g., patients, clients, members, customers)	34
People who are affected themselves as well as relatives	8
Relatives (e.g., relatives association)	1
Other (e.g., member organizations, employers, health professionals)	10
Total	53

#### Descriptive analyses

The mean per scale and the distribution of the means per organization are presented in Table 4. The first scale (“easy access and navigation”) shows the highest mean (*M =* 3.1). On average, items belonging to this scale were at that point being implemented more than “mostly”. The lowest mean can be found for the fifth scale (“involvement and support of target groups”) (*M = 2.3).* Missing values per item varied a lot, ranging from no missing value up to 43.4% missing values in one item of the fifth subscale (Additional file 2). Most missing values can be attributed to not being applicable to the respective organization.

Analyses also revealed large differences between the organizations’ individual means. While the first scale showed the smallest range of mean values (lowest mean value 1.3, highest mean value 3.9), the fifth scale had the largest range with mean values from 0.0 to 4.0 (see Table 4).

**Table 4:** Descriptive results by scale

Scale (n cases)	Mean	Missings (in %)*	Distribution of mean values per organization		
			Range	SD**	Median (25%; 75%)
1) Easy access and navigation (53)	3.1	0.0-20.8	1.3–3.9	0.6	3.2 (2.8; 3.4)
2) Integration, prioritization, and dissemination of OHL (50)	2.7	3.8–24.5	0.5-4.0	0.9	3.0 (2.3; 3.3)
3) Qualification, quality management, evaluation, and needs assessment (52)	2.5	3.8–34.0	0.7-4.0	1.0	2.7 (1.6; 3.3)
4) Communication with target groups (52)	2.8	7.6–22.6	0.0-3.9	0.8	2.9 (2.5; 3.4)
5) Involvement and support of target groups (47)	2.3	22.6–43.4	0.0–4.0	1.0	2.4 (1.6; 3.0)

Response scale: 0 “not at all”, 1 “a little”, 2 “somewhat/partly”, 3 “mostly”, 4 “completely”

* Including “not applicable”; ** SD = standard deviation

#### Psychometric analyses

Table 5 shows the psychometric properties of the five scales of the self-assessment tool. Results on item levels are summarized by showing the range of the parameters for each item per scale. Details about the distribution of answers per item are shown in the appendix (Additional file 2). Furthermore, the full description of missing values, mean, standard deviation, item difficulty, item discrimination, and Cronbach’s alpha can be found in Additional file 3. There were significant flooring effects in one scale only (“involvement and support of target groups”). None of the scales showed significant ceiling effects. We found good to excellent values for internal consistency for each subscale, indicated by Cronbach’s alpha (“easy access and navigation” = 0.87, “integration, prioritization, and dissemination of OHL” = 0.93, “qualification, quality management, evaluation, and needs assessment” = 0.95, “communication with target groups” = 0.95, and “involvement and support of target groups” = 0.81).

No item of the five scales showed flooring effects. In four scales we found items with ceiling effects (five items in scale “easy access and navigation”, two items in scale “qualification, quality management, evaluation, and needs assessment”, five items in scale “communication with target groups”, and one item in scale “involvement and support of target groups”). All scales showed items with either too low or too high item discrimination. “Easy access and navigation” had 11 items with satisfactory and five items with lower or higher item discrimination. The second scale “integration, prioritization, and dissemination of OHL” contained five satisfactory items, while six items showed higher discrimination. Item discrimination for “qualification, quality management, evaluation, and needs assessment” was satisfactory in nine out of 17 items. Eight items had higher discrimination values. For the fourth scale “communication with target groups”, we found 10 items with satisfactory discrimination, while 15 items either showed lower or higher discrimination. The fifth scale “involvement and support of target groups” contains four satisfactory items and four items with lower or higher discrimination.

**Table 5:** Psychometric properties of the self-assessment tool on scale and item levels

Scale (N items)	Scale level measures			Summary of item-level measures	
	Flooring (in %)	Ceiling (in %)	Cron-bach’s α	Item Difficulty	Item Discrimination
1) Easy access and navigation (16)	0.0	9.4	0.87	0.54–0.91	0.22–0.75
2) Integration, prioritization, and dissemination of OHL (11)	7.5	7.5	0.93	0.51–0.78	0.50–0.91
3) Qualification, quality management, evaluation, and needs assessment (17)	7.5	3.8	0.95	0.31–0.88	0.42–0.90
4) Communication with target groups (25)	3.8	3.8	0.95	0.40–0.93	0.31–0.81
5) Involvement and support of target groups (8)	15.1*	0.0	0.81	0.31–0.81	-0.04-0.82

* Effects were considered significant if the proportion of floor or ceiling effects exceeded 15%

## Discussion

### Summary of findings

The study described the development and testing of a self-assessment tool for measuring OHL. The development process was based on multiple methods and started with a scoping review extracting criteria that characterize health literate health care organizations formerly published by the authors [14]. Group discussions with health care organizations were held to supplement the criteria identified by the scoping review. The synthesized pool of criteria was reduced to a selection of unique criteria by paraphrasing and summarizing criteria. This first draft of the self-assessment tool underwent further adaptions using a Delphi study. The Delphi study did not reveal a pattern of differing importance by type of organization. Instead, the feedback underlined the perceived importance of all the criteria applicable to the respective organization. In addition, a review by the project’s patient advisory council was included in the process. Further changes to the tool were applied following a pretest and a final internal revision.

The detailed development process finally identified 77 OHL items that were grouped into 17 subscales and the following five scales: (1) “easy access and navigation”, (2) “integration, prioritization, and dissemination of organizational health literacy”, (3) “qualification, quality management, evaluation, and needs assessment”, (4) “communication with target groups”, and (5) “involvement and support of target groups”.

The online survey showed the highest mean OHL score for “easy access and navigation” whereas “involvement and support of target groups” had the lowest mean value. Regarding psychometric properties on a scale level, results showed good to excellent internal consistency of the five scales, indicated by high Cronbach’s alpha values. Furthermore, only one scale had significant flooring effects, while no ceiling effects were found on a scale level. On the item level, the analysis of item difficulty showed no flooring effects, while 13 out of 77 items had ceiling effects. 39 Items had satisfactory item discrimination, while discrimination for the remaining 38 items was either below or above the desirable range.

### Interpretation of findings

As the development of the tool was based on criteria extracted from already existing tools as well as other types of publications [14] and was supplemented with further criteria identified in group discussions, the tool includes more aspects of OHL than individual tools taken into account (e.g., [6, 12, 27]). In total, our new self-assessment tool for measuring OHL consists of five scales, the diverse aspects of which are specified in 77 items. Therefore, we offer a more detailed tool for measuring OHL compared to, for example, tools [6] primarily based on the field shaping 10 attributes by Brach et al. [1]. This allows the self-assessment tool to be used by a wide range of types of health care organizations as demonstrated with the pilot test. Therefore, this study adds further possibilities to analyze OHL beyond the existing tools that primarily address or have been tested in the context of specific types of organizations such as, for example, hospitals [5–7], pharmacies [8], primary care practices [9–12], or organizations belonging to the community sector [13]. Our tool is based on a broad combination of multiple methods as opposed to other tools that were derived from development processes with fewer stages (e.g., [5, 28]). In addition, the multi-staged development process of the OHL-HAM tool involved service users – a group that, according to our knowledge, has not yet been extensively involved with the development or testing processes of other tools [10, 11, 17, 29, 30].

We found good to excellent results regarding internal consistency, indicating that the items are well assigned to our five scales, which were based on the scoping review and the qualitative analysis of the goup discussions. This is in contrast to studies from Kowalski et al. [6] or Altin et al. [9] that validated one-dimensional questionnaire structures to measure OHL. However, this is not surprising as our self-assessment tool consists of more items compared to the more general, limited set of criteria selected in the before-mentioned studies, which only used 10 or four items, respectively. Most likely the reason for the opposing results is that our self-assessment tool captures a more differentiated picture of OHL. This approach is in line with other research that developed and validated questionnaires covering different domains of OHL [31]. Nonetheless, it is important to further validate our tool in larger studies. In this context, we would recommend refining the scales to reduce the number of items and to improve the content consistency of the scales. This is also supported by the results of the item analysis that some items need to be removed from the scale to improve internal consistency. For example, the item “Target groups of the organization are represented in a board that actively advises and helps shape the organization.” of the fifth scale has a negative item discrimination. A reason might be that this item does not represent how organizations involve target groups. A larger sample is required to enable such a detailed investigation of psychometric properties. Exclusion of inappropriate items would further improve discrimination and thus the quality of each scale concerning OHL.

### Strengths and limitations

Profound literature research supplemented with group discussions among representatives of various health care contexts formed the base of the developed OHL self-assessment tool. The accompanying Delphi study did not identify any organization type-specific patterns and only lead to small adaptions of the gathered criteria. Therefore, the resulting tool consists of a large number of items that could not be further reduced at this stage of the process. The length of the tool may have resulted in a lower willingness to participate in the survey. Simultaneously, the scope of the tool underlines its potential to capture the various aspects of OHL. In comparison to OHL tools with broader criteria, the more detailed items of the presented tool might be easier for organizations to evaluate and implement. A large number of items can also be attributed to the aim of making the tool usable for different types of organizations which increases the diversity of topics and areas of application.

Testing the tool was an important first step in examining the structure of the questionnaire. The psychometric analysis supported the theoretical assumption of a five-scale self-assessment tool with good item loadings and item difficulties, as well as convincing reliability of the five scales. Unlike the previously mentioned studies that represent OHL one-dimensionally, the self-assessment tool presented here can be an improvement to adequately measure OHL as a multidimensional concept. Nevertheless, this pilot study was just explorative with a small sample size of 53 cases. Furthermore, we cannot rule out that participants probably had a biased perception regarding the extent to which OHL is established in their organizations. Collecting data from employees working in different positions could have helped to get a more realistic idea. Therefore, the results are non-representative of the Hamburg region, and further validation of the self-assessment tool is required. Additionally, the participating organizations were recruited in the metropolitan area of Hamburg and thus are likely to differ from health care organizations located in rural areas. For example, specialized services or organizations embedded in more developed co-operation networks could be overpresented in our sample. While some of these factors certainly have an impact on the quality of and access to health care, they could also affect the degree to which organizations are engaged in OHL. The psychometric analysis would probably benefit from broader sampling strategy due to the larger variation in the data.

We conducted a multi-staged development process for the OHL self-assessment tool. The process was literature- and consensus-based providing the tool with a broad empirical background. Next to involving representatives of various types of organizations, the study incorporated feedback from the project’s patient advisory council. Taking both perspectives from the heterogeneous provider and user side into consideration is crucial for the field of OHL and strengthens the basis of the developed tool.

## Conclusions

Increasing OHL helps to remove barriers and improves the provision of health care for everyone. Therefore, the evaluation and implementation of OHL in health care organizations can be an important contribution to improving the health care system. Overall, we were able to identify a set of 77 criteria, which characterize a health literate organization. With this, we can provide a newly developed and broadly empirically based OHL self-assessment tool. Health care organizations can use this self-assessment tool to identify aspects to work on to improve their health literacy. The psychometric analysis showed a high internal consistency for all scales as well as good item loadings for the different items on the five scales. A further study with a bigger sample size is needed to check the validity. Reducing the number of items could also be relevant in the further development of the self-assessment tool. Based on the large set of items, a future self-assessment tool could be adjusted and validated for different types of health care organizations. In particular, a shorter version of the self-assessment tool would be desirable, as it would be easier to use for the health care organizations, as well as research purposes.

### Electronic supplementary material

Below is the link to the electronic supplementary material.

**Additional file 1**: Final OHL self-assessment tool

**Additional file 2**: Item distribution

**Additional file 3**: Reliability

## Data Availability

The dataset generated and analyzed during the study are available in the OSF repository, https://osf.io/xsh3f/ (doi 10.17605/OSF.IO/XSH3F). The study protocol, search syntax, checklist, list of extractions, and included and excluded studies are provided as additional files to the original publication of the scoping review [14]. A study protocol including all preliminary specifications is accessible on OSF Registries [32]. The English version of the items that form the self-assessment tool can be found in Additional file 1. Please note that the English version has not been tested yet and might be subject to later rephrasing since the original research was conducted in German.

## Abbreviations

OHL: Organizational health literacy

## References

[1] Brach C, Keller D, Hernandez LM, Baur C, Parker R, Dreyer B, Schyve P, Lemerise AJ, Schillinger D. (2012) Ten attributes of health literate health care organizations.

[2] Sørensen K, van den Broucke S, Fullam J, Doyle G, Pelikan JM, Slonska Z, Brand H (2012). Health literacy and public health: a systematic review and integration of definitions and models. BMC Public Health.

[3] Pelikan JM (2017). Gesundheitskompetente Krankenbehandlungseinrichtungen. Public Health Forum.

[4] International Working Group Health Promoting Hospitals and Health Literate Healthcare Organizations (Working Group HPH & HLO). (2019) International Self-Assessment Tool Organizational Health Literacy (Responsiveness) for Hospitals - SAT-OHL-Hos-v1.1-EN-international.

[5] Pelikan JM, Dietscher C. (2015) Developing the organizational health literacy of health organizations: Strategies and examples [Die Gesundheitskompetenz von Gesundheitseinrichtungen entwickeln: Strategien und Beispiele].

[6] Kowalski C, Lee S-YD, Schmidt A, Wesselmann S, Wirtz MA, Pfaff H, Ernstmann N (2015). The health literate health care organization 10 item questionnaire (HLHO-10): development and validation. BMC Health Serv Res.

[7] Rudd RE, Anderson JE. (2006) The health literacy environment of hospitals and health centers.

[8] Jacobson KL, Gazmararian JA, Kripalani S, McMorris KJ, Blake SC, Brach C. (2007) Is our pharmacy meeting patients’ needs? A pharmacy health literacy assessment tool user’s guide.

[9] Altin SV, Lorrek K, Stock S (2015). Development and validation of a brief screener to measure the health literacy responsiveness of Primary Care practices (HLPC). BMC Fam Pract.

[10] Stuermer N, De Gani SM, Beese A-S, Giovanoli Evack J, Jaks R, Nicca D (2022). Health professionals’ experience with the first implementation of the Organizational Health literacy Self-Assessment Tool for primary care (OHL Self-AsseT)—A qualitative reflexive thematic analysis. Int J Environ Res Public Health.

[11] de Gani SM, Nowak-Flück D, Nicca D, Vogt D (2020). Self-assessment tool to promote Organizational Health Literacy in primary care settings in Switzerland. Int J Environ Res Public Health.

[12] DeWalt DA, Callahan LF, Hawk VH, Broucksou KA, Hink A, Rudd RE, Brach C. (2010) Health Literacy Universal Precautions Toolkit. 10.1016/j.outlook.2010.12.002 PMC5091930 21402204

[13] Tasmanian Council of Social Service (TasCOSS). (2020) HeLLO Tas! a toolkit for health literacy learning organisations.

[14] Bremer D, Klockmann I, Jaß L, Härter M, von dem Knesebeck O, Lüdecke D (2021). Which criteria characterize a health literate health care organization? - a scoping review on organizational health literacy. BMC Health Serv Res.

[15] Xyländer M, Kleineke V, Jünger S (2020). Group Discussions in Health Services Research – part 2: reflections on the Concept of Group, Moderation and Analysis of Group Discussions as well as Online Group Discussi-on [Gruppendiskussionen in Der Versorgungsforschung – Teil 2: Überlegungen Zum Begriff Der Gruppe, Zur Moderation Und Auswertung Von Gruppendiskussionen Sowie Zur Methode Der Online-Gruppendiskussion]. Gesundheitswesen.

[16] Pohontsch NJ, Müller V, Brandner S (2018). Group Discussions in Health Services Research – part 1: introduction and deliberations on selection of Method and Planning [Gruppendiskussionen in Der Versorgungsforschung – Teil 1: Einführung Und Überlegungen Zur Methodenwahl Und Planung]. Gesundheitswesen.

[17] Trezona A, Dodson S, Osborne RH (2017). Development of the organisational health literacy responsiveness (Org-HLR) framework in collaboration with health and social services professionals. BMC Health Serv Res.

[18] Mayring P (2015). Qualitative content analysis: basics and techniques [Qualitative inhaltsanalyse: Grundlagen Und Techniken].

[19] Fitch K, Bernstein SJ, Aguilar MD, Burnand B, LaCalle JR, Lazaro P, van het Loo M, McDonnell J, Vader J, Kahan JP (2001). The RAND/UCLA appropriateness method user’s manual.

[20] German Federal Ministry of Health. (2022) Our health care system [Unser Gesundheitssystem].

[21] Rodrigues IB, Adachi JD, Beattie KA, Lau A, MacDermid JC. (2019) Determining known-group validity and test-retest reliability in the PEQ (personalized exercise questionnaire). BMC musculoskeletal disorders. 10.1186/s12891-019-2761-3. 10.1186/s12891-019-2761-3 PMC6694546 31412834

[22] Terwee CB, Bot SDM, de Boer MR, van der Windt DAWM, Knol DL, Dekker J, Bouter LM, de Vet HCW (2007). Quality criteria were proposed for measurement properties of health status questionnaires. J Clin Epidemiol.

[23] Kelava A, Moosbrugger H, Moosbrugger H, Kelava A (2020). Descriptive statistical item analysis and test score determination [Deskriptivstatistische Itemanalyse Und Testwertbestimmung]. Testtheorie Und Fragebogenkonstruktion.

[24] Bland JM, Altman DG (1997). Cronbach’s alpha. BMJ (Clinical Research ed).

[25] Lüdecke D, Ben-Shachar M, Patil I, Waggoner P, Makowski D (2021). Performance: an R package for assessment, comparison and testing of statistical models. J Open Source Softw.

[26] Lüdecke D, Ben-Shachar M, Patil I, Makowski D (2020). Extracting, computing and exploring the parameters of statistical models using R. J Open Source Softw.

[27] NALA (2009). Literacy audit for healthcare settings.

[28] Dietscher C, Pelikan JM (2016). Health-literate healthcare organizations: feasibility study of organizational self-assessment with the Vienna tool in Austrian hospitals [Gesundheitskompetente Kranken-behandlungsorganisationen: Machbarkeitsstudie Zur Organisationalen Selbstbewertung Mit dem Wiener Instrument in österreichischen Krankenhäusern]. Präv Gesundheitsf.

[29] Trezona A, Dodson S, Osborne RH (2018). Development of the Organisational Health Literacy Responsiveness (Org-HLR) self-assessment tool and process. BMC Health Serv Res.

[30] Trezona A, Dodson S, Fitzsimon E, LaMontagne AD, Osborne RH (2020). Field-testing and refinement of the Organisational Health literacy responsiveness self-assessment (Org-HLR) tool and process. Int J Environ Res Public Health.

[31] Weidmer BA, Brach C, Hays RD (2012). Development and evaluation of CAHPS survey items assessing how well healthcare providers address health literacy. Med Care.

[32] Klockmann I, Lüdecke D, Härter M, von dem Knesebeck O, Bremer D. (2021) Which criteria characterize a health literate healthcare organization? – A scoping review protocol on organizational health literacy. 10.17605/OSF.IO/W9TUP. 10.1186/s12913-021-06604-z PMC8259028 34229685

