# Supplementary material for: Multi-staged development and pilot testing of a self-assessment tool for organizational health literacy
Source: BMC Health Serv Res. 2023 Dec 13;23:1407. doi: 10.1186/s12913-023-10448-0 (PMC10720162; doi:10.1186/s12913-023-10448-0)
Supplement: Supplementary file 3 — Additional file 3: Reliability [file 12913_2023_10448_MOESM3_ESM.docx]

**Reliability**

Table 1: Scale 1 – Easy access and navigation

| **Item** | **Missings (in %)** | **Mean** | **Standard Deviation** | **Item Difficulty** | **Item Discrimination** | **α if deleted** |
| --- | --- | --- | --- | --- | --- | --- |
| Information on directions to and orientation within the organization is easy to find and understand. | 3.77 | 3.25 | 0.82 | 0.81 | 0.57 | 0.86 |
| Telephone contact with the organization is free of charge and available at flexible times. | 1.89 | 3.02 | 1.13 | 0.75 | 0.22 | 0.88 |
| Telephone contact with the organization is informative and handled by competently trained staff. | 0.00 | 3.40 | 0.79 | 0.85 | 0.24 | 0.88 |
| The online presence of the organization allows relevant information to be found by using a clear structure (e.g., by using links) and can be navigated without barriers (e.g., with a screen reader). | 5.66 | 2.86 | 1.03 | 0.72 | 0.44 | 0.87 |
| Entry to the organization is barrier-free. | 7.55 | 3.02 | 1.38 | 0.76 | 0.41 | 0.87 |
| Financial barriers regarding the use of services are reduced and the financial capabilities of users are taken into account. | 20.75 | 3.33 | 1.00 | 0.83 | 0.57 | 0.86 |
| A (professionally) comprehensive range of services and treatments within the organization ensures adequate care for the users. | 13.21 | 3.17 | 1.06 | 0.79 | 0.49 | 0.87 |
| In order to be able to reach certain target groups and, for example, to recommend certain services or consultations to them, individual direct contact is made with users in suitable and necessary situations. | 7.55 | 3.10 | 1.07 | 0.78 | 0.52 | 0.87 |
| The navigation within the organization is facilitated by easy-to-understand signage, navigational aids, and further information. | 16.98 | 2.80 | 1.07 | 0.70 | 0.39 | 0.87 |
| The staff helps users to find their way around the organization. | 15.09 | 3.62 | 0.89 | 0.91 | 0.65 | 0.86 |
| The staff helps users to apprehend the range of offered services and, for example, to fill in forms correctly. | 16.98 | 3.61 | 0.78 | 0.90 | 0.72 | 0.86 |
| The rooms are adapted to the individual needs of users (e.g., privacy) and to the type of services that are offered by the organization. | 13.21 | 3.20 | 1.07 | 0.80 | 0.66 | 0.86 |
| Referrals to other health organizations (e.g., medical specialists, nutrition counseling) are facilitated, supported, or directly handled. | 7.55 | 3.18 | 0.95 | 0.80 | 0.62 | 0.86 |
| Referrals to non-medical support services (e.g., mobility or food services, or other community services) are facilitated, supported, or directly handled. | 16.98 | 2.82 | 1.23 | 0.70 | 0.72 | 0.85 |
| Information about the organization's services as well as services beyond those of the organization (e.g., emergency medical service, city service portals) is spread by public relations work (e.g., using advertisements, trade fairs, or yellow pages). | 13.21 | 2.15 | 1.28 | 0.54 | 0.46 | 0.87 |
| To reach, educate and provide care for the target groups, cooperations with other health organizations (e.g., regarding referrals or follow-up examinations) and further organizations such as schools, daycare facilities for children, and businesses are established. | 15.09 | 3.00 | 1.11 | 0.75 | 0.60 | 0.86 |

Table 2: Scale 2 – Integration, prioritization, and dissemination of OHL

| **Item** | **Missings (in %)** | **Mean** | **Standard Deviation** | **Item Difficulty** | **Item Discrimination** | **α if deleted** |
| --- | --- | --- | --- | --- | --- | --- |
| The organization is dedicated to its organizational health literacy and embeds the concept in its mission statement across all organizational levels. | 13.21 | 2.87 | 1.19 | 0.72 | 0.81 | 0.92 |
| Leadership actively supports the work on organizational health literacy. | 15.09 | 3.13 | 1.10 | 0.78 | 0.82 | 0.92 |
| Guidelines, initiatives, and measures for the implementation of health literacy are resolved and implemented. | 15.09 | 2.64 | 1.19 | 0.66 | 0.71 | 0.92 |
| A health literacy promoting structure and environment is created. | 11.32 | 2.7 | 1.23 | 0.68 | 0.91 | 0.91 |
| With the involvement of staff, the organization is dedicated to a continuous process of development. | 7.55 | 3.04 | 1.00 | 0.76 | 0.65 | 0.93 |
| Staff resources are provided. | 16.98 | 2.52 | 1.05 | 0.63 | 0.73 | 0.92 |
| Personnel responsibilities and accountabilities for the implementation of organizational health literacy are clarified and assigned in a binding manner. | 15.09 | 2.44 | 1.32 | 0.61 | 0.69 | 0.92 |
| Within the organization, resources (e.g., rooms, equipment, and technology), tools, and processes for the practical implementation of health literacy are developed and/or made available. | 16.98 | 2.39 | 1.30 | 0.60 | 0.79 | 0.92 |
| Political and legal requirements applicable to the organization (e.g., UN Convention on the Rights of Persons with Disabilities) are taken into account or implemented whilst implementing organizational health literacy. | 22.64 | 2.95 | 1.02 | 0.74 | 0.50 | 0.93 |
| Organizational health literacy is promoted beyond the context of the own organization and is shaped in cooperation with others. | 22.64 | 2.12 | 1.19 | 0.53 | 0.58 | 0.93 |
| The organization undertakes or supports research in the field of individual and organizational health literacy. | 18.87 | 2.02 | 1.41 | 0.51 | 0.64 | 0.93 |

Table 3: Scale 3 – Qualification, quality management, evaluation, and needs assessment

| **Item** | **Missings (in %)** | **Mean** | **Standard Deviation** | **Item Difficulty** | **Item Discrimination** | **α if deleted** |
| --- | --- | --- | --- | --- | --- | --- |
| The knowledge and importance of organizational health literacy are promoted and cultivated. | 15.09 | 2.40 | 1.14 | 0.60 | 0.70 | 0.95 |
| The staff is qualified in techniques for communication with target groups. | 11.32 | 2.55 | 1.28 | 0.64 | 0.57 | 0.95 |
| Social as well as emotional competencies and respectful interaction with users are promoted, for example, by respecting their privacy. | 11.32 | 3.53 | 0.86 | 0.88 | 0.61 | 0.95 |
| Clear, effective, and open communication among staff is encouraged within the organization. | 3.77 | 3.41 | 0.92 | 0.85 | 0.42 | 0.95 |
| Knowledge about and the importance of individual health literacy is promoted and cultivated. | 11.32 | 3.04 | 1.10 | 0.76 | 0.67 | 0.95 |
| The staff's individual health literacy is fostered. | 9.43 | 2.92 | 1.07 | 0.73 | 0.66 | 0.95 |
| Quality management of organizational health literacy is pursued. | 24.53 | 1.90 | 1.45 | 0.48 | 0.61 | 0.95 |
| Goals for improving organizational health literacy are defined and responsibilities for achieving these goals are established. | 24.53 | 1.82 | 1.28 | 0.46 | 0.80 | 0.94 |
| Appropriate tools (e.g., (self-)assessment tools) are used to evaluate the pursuit of organizational health literacy goals. | 24.53 | 1.40 | 1.43 | 0.35 | 0.78 | 0.94 |
| Already existing as well as developed materials, programs, policies, training, and the overall service environment are reviewed and evaluated against the goals defined for improving organizational health literacy. | 22.64 | 1.71 | 1.21 | 0.43 | 0.90 | 0.94 |
| Feedback from target groups is collected and processed to evaluate the defined goals. | 16.98 | 2.07 | 1.50 | 0.52 | 0.86 | 0.94 |
| The needs of the target groups are surveyed and it is evaluated how well these needs are addressed. | 15.09 | 2.13 | 1.38 | 0.53 | 0.66 | 0.95 |
| Users are given the opportunity to contact a complaints body of the organization. | 18.87 | 2.95 | 1.41 | 0.74 | 0.55 | 0.95 |
| Staff knowledge about organizational health literacy is evaluated. | 22.64 | 1.24 | 1.32 | 0.31 | 0.84 | 0.94 |
| Staff is actively involved in the further development of materials and the organization as a whole. | 13.21 | 2.28 | 1.46 | 0.57 | 0.83 | 0.94 |
| Internal experts on individual and organizational health literacy are consulted for the development of measures and materials. | 20.75 | 1.95 | 1.56 | 0.49 | 0.82 | 0.94 |
| External experts on individual and organizational health literacy are consulted for the development of measures and materials. | 24.53 | 1.35 | 1.39 | 0.34 | 0.67 | 0.95 |

Table 4: Scale 4 – Communication with target groups

| **Item** | **Missings (in %)** | **Mean** | **Standard Deviation** | **Item Difficulty** | **Item Discrimination** | **α if deleted** |
| --- | --- | --- | --- | --- | --- | --- |
| Easy-to-understand language is used in direct conversation and other oral communication with target groups (e.g., telephone, video). | 3.77 | 3.14 | 0.87 | 0.78 | 0.56 | 0.95 |
| Easy-to-understand language is used in discharge plans, information brochures, and other materials. | 11.32 | 2.70 | 1.02 | 0.68 | 0.64 | 0.95 |
| Layouts, visualizations, symbols, and for example, font formatting are designed to be easy to understand and barrier-free. | 5.66 | 2.56 | 1.13 | 0.64 | 0.64 | 0.95 |
| Services and information are provided in foreign languages and, if necessary, translation services or technical aids (e.g., online text translation or interpretation) are used. | 11.32 | 1.91 | 1.47 | 0.48 | 0.38 | 0.95 |
| The education of target groups follows a systematic and targeted strategy that has been resolved for the organization. | 18.87 | 2.86 | 1.17 | 0.72 | 0.57 | 0.95 |
| Health-related information is made easily accessible and free of charge to the target groups. | 7.55 | 3.02 | 1.05 | 0.76 | 0.73 | 0.95 |
| The organization informs, educates, and offers assistance regarding a variety of topics that lie within the organization's respective range of action (e.g., prevention, diagnostics, and therapy as well as social and financial matters). | 7.55 | 3.33 | 0.90 | 0.83 | 0.76 | 0.95 |
| The organization provides health-related learning and education (e.g., preventive education) for its target groups. | 15.09 | 2.80 | 1.20 | 0.70 | 0.80 | 0.95 |
| The individual health literacy of users is fostered. | 13.21 | 2.91 | 1.15 | 0.73 | 0.82 | 0.95 |
| The individual health literacy of relatives (e.g., family) is fostered. | 33.96 | 2.49 | 1.34 | 0.62 | 0.71 | 0.95 |
| Target groups are linked to internal and external educational resources and services. | 20.75 | 2.36 | 1.25 | 0.59 | 0.72 | 0.95 |
| Communication with target groups is transparent (e.g., information on cost coverage). | 9.43 | 3.71 | 0.62 | 0.93 | 0.61 | 0.95 |
| The content is reliable, up-to-date, offers precise recommendations for action, and meets scientific standards. | 7.55 | 3.67 | 0.63 | 0.92 | 0.55 | 0.95 |
| Tools (e.g., accessibility checking software) are used for the creation of print and online materials to support the creation of easy-to-understand materials. | 33.96 | 1.71 | 1.25 | 0.43 | 0.44 | 0.95 |
| A range of different formats, channels, and media (e.g., social media, video clips) are specifically used for external communication with target groups. | 18.87 | 2.56 | 1.10 | 0.64 | 0.31 | 0.95 |
| The technology used is designed in such a way that it is easy for the target groups to understand and use without barriers. | 20.75 | 2.55 | 0.97 | 0.64 | 0.60 | 0.95 |
| The organization takes the diversity of its target groups into account, for example, in terms of cultural and religious backgrounds, and is dedicated to inclusion and equality for all. | 9.43 | 3.21 | 0.94 | 0.80 | 0.39 | 0.95 |
| Amongst the staff, awareness is created on how the interaction with health organizations is perceived by users and what influence the users' individual health literacy has in this regard. | 22.64 | 2.93 | 1.10 | 0.73 | 0.46 | 0.95 |
| Staff avoids making assumptions about the general level of knowledge and individual health literacy of users. | 15.09 | 3.24 | 1.03 | 0.81 | 0.57 | 0.95 |
| The organization is committed to destigmatizing illness and utilization of care services. | 9.43 | 3.48 | 1.05 | 0.87 | 0.88 | 0.95 |
| Different communication needs, barriers, and preferences of users are acknowledged (e.g., in terms of individual health literacy, language, and culture) and communication and interaction are adapted accordingly. | 15.09 | 2.87 | 1.24 | 0.72 | 0.84 | 0.95 |
| Ensurance of understanding is defined as part of the communication culture and standard that is followed during every contact with users, especially in risk situations such as consent to surgical interventions. | 32.08 | 2.69 | 1.35 | 0.67 | 0.86 | 0.95 |
| Different methods are used to check whether the users have correctly understood what has been explained, to encourage them to ask questions, and, if necessary, further information is explained in an easy-to-understand way. | 24.53 | 2.72 | 1.15 | 0.68 | 0.91 | 0.95 |
| Difficulties and errors in communication are systematically processed, dealt with, and evaluated as a risk factor for users. | 30.19 | 1.59 | 1.38 | 0.40 | 0.72 | 0.95 |
| Communication principles, such as ensuring understanding or dealing with communication difficulties, become part of the routine and are also followed in high-risk situations. | 33.96 | 2.37 | 1.24 | 0.59 | 0.86 | 0.95 |

Table 5: Scale 5 – Involvement and support of target groups

| **Item** | **Missings (in %)** | **Mean** | **Standard Deviation** | **Item Difficulty** | **Item Discrimination** | **α if deleted** |
| --- | --- | --- | --- | --- | --- | --- |
| Target groups are actively involved in the development of materials and services. | 22.64 | 1.44 | 1.03 | 0.36 | 0.53 | 0.79 |
| Relatives (e.g., family) are actively involved in the development of materials and services. | 41.51 | 1.23 | 1.28 | 0.31 | 0.46 | 0.80 |
| Target groups are included in care processes and conversations. | 26.42 | 2.54 | 1.33 | 0.63 | 0.57 | 0.78 |
| Target groups of the organization are represented in a board that actively advises and helps shape the organization. | 33.96 | 1.37 | 1.46 | 0.34 | -0.04 | 0.85 |
| The role of caregivers in supporting the users is considered important by the organization. | 32.08 | 3.25 | 1.16 | 0.81 | 0.67 | 0.77 |
| Users are supported in various areas of self-management (e.g., taking medication, exercise, nutrition) beyond the duration of treatment. | 41.51 | 2.52 | 1.43 | 0.63 | 0.77 | 0.74 |
| In order to support the self-management of users, different methods are used, for example, an action plan is drawn up or follow-up appointments are held to check the use of medication. | 43.40 | 2.53 | 1.55 | 0.63 | 0.82 | 0.73 |
| The organization offers space for peer-to-peer approaches and opportunities for exchange in groups (e.g., self-help groups), which serve the mutual empowerment of members. | 26.42 | 2.13 | 1.47 | 0.53 | 0.39 | 0.81 |
